# 1229. Outpatient Penicillin and Sulfonamide Antibiotic Allergy E-Consult Service in Prospective Solid Organ Transplant Recipients

**DOI:** 10.1093/ofid/ofad500.1069

**Published:** 2023-11-27

**Authors:** Amanda M Bair, Shyam Joshi, Karen Anstey, Amber C Streifel, YoungYoon Ham

**Affiliations:** Oregon Health & Science University (OHSU), Portland, Oregon; Oregon Health & Science University, Portland, Oregon; OHSU, Portland, Oregon; Oregon Health and Science University, Portland, Oregon; Oregon Health & Science University, Portland, Oregon

## Abstract

**Background:**

Solid organ transplant recipients (SOTr) are at increased risk for infections post-transplantation. A penicillin allergy incidence of 12% and sulfonamide antibiotic allergy incidence of 9% has been reported in transplant recipients. Documented penicillin and sulfonamide allergies lead to the unnecessary use of broad-spectrum antibiotics or suboptimal therapy leading to further adverse consequences. The purpose of this study is to improve the rates of antibiotic allergy delabeling in SOTr prior to transplantation through the implementation of an allergy testing pilot program.

**Methods:**

From December 15, 2022 to April 28, 2023, prospective SOTr with a penicillin and/or sulfonamide allergy that was tested and delabeled at OHSU were included in this study. This process involves a clinical pharmacist contacting the patient and collecting a detailed allergy history via an e-consult. The e-consult is then routed to one of our allergists to determine patient testing eligibility. If the patient qualifies for testing, they are scheduled for a nurse visit at the clinic. After testing is completed, patients are notified of their results and the allergy is removed from their electronic health record with accompanying documentation.

**Results:**

A total of 86 patients were evaluated for allergy testing (61% penicillin allergies and 39% sulfonamide allergies). Fourteen patients were successfully delabeled through the pilot program with one of the patients having both a penicillin and sulfonamide allergy delabeled. The primary reason for patients being excluded from this study was due to insurance not covering allergy testing done at OHSU as shown in Table 1. Although not included in the pilot program, 7 patients were successfully delabeled by obtaining a detailed allergy history and did not require allergy testing. Twenty-two patients have a future allergy testing appointment scheduled.

**Table 1:**
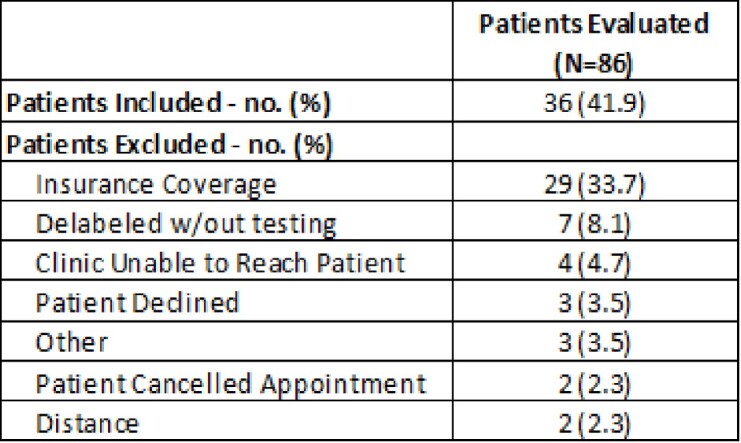
Patient Inclusion and Exclusion

**Conclusion:**

The preliminary results of our study show the implementation of an e-consult service for antibiotic allergy testing in prospective SOTr prior to transplantation is an effective method for allergy delabeling. Allergy testing in this patient population has the potential to significantly improve the selection of antibiotics available for treatment as well as prophylaxis.

**Disclosures:**

**Shyam Joshi, MD**, Cogent: Honoraria|Leo Pharma: Honoraria|Nectar Allergy: Advisor/Consultant|Nectar Allergy: Stocks/Bonds|Sanofi/Regeneron: Advisor/Consultant|Sanofi/Regeneron: Honoraria|Takeda: Honoraria

